# Self-Supervised Hierarchical Dilated Transformer Network for Hyperspectral Soil Microplastic Identification and Detection

**DOI:** 10.3390/s25216517

**Published:** 2025-10-22

**Authors:** Peiran Wang, Xiaobin Li, Ruizhe Zhang, Qiongchan Gu, Lianchi Zhang, Jiangtao Lv

**Affiliations:** 1 School of Intelligent Sensing and Optoelectronic Engineering, Northeastern University at Qinhuangdao, Qinhuangdao 066004, China; 2 Hebei Key Laboratory of Micro-Nano Precision Optical Sensing and Measurement Technology, School of Control Engineering, Northeastern University at Qinhuangdao, Qinhuangdao 066004, China; 3 Water Resources and Water Conservation Development Center of Chengde, Chengde 067000, China; 4 College of Information Science and Engineering, Northeastern University, Shenyang 110004, China; 5 FAW Car Co., Ltd., Changchun 130012, China

**Keywords:** environmental monitoring, soil microplastics, hyperspectral classification, contrastive learning, transformer network

## Abstract

Microplastics are plastic particles less than five millimeters in diameter that have led to serious environmental problems, and detecting these tiny particles is crucial to understanding their distribution and impact on the soil environment. In this paper, we propose the Self-Supervised Hierarchical Dilated Transformer Network (SHDTNet), an improved hyperspectral image classification model based on self-supervised contrastive learning, for identifying and detecting microplastics in soil. Currently, most hyperspectral image classifications rely on supervised methods, which perform well with rich training samples. However, pixel labeling in soil microplastic detection scenarios is a difficult and costly task. By employing the self-supervised contrastive learning technique, SHDTNet addresses the problem of insufficient training samples for hyperspectral images of soil microplastics and also enhances the feature extraction module in contrastive learning to improve the network model’s feature extraction capability. Experiments on self-constructed hyperspectral soil microplastic image datasets demonstrate that the proposed method accurately recognizes unique microplastics in the soil environment without errors or missed detections, outperforming several currently available soil microplastic detection methods.

## 1. Introduction

Plastic particles in the environment that are smaller than five millimeters in diameter or length are referred to as microplastics (MPs) [1]. Microplastics (MPs) are now pervasive in the environment. They are persistent and latent, contributing to pollution in soil, oceans, and the atmosphere [2,3,4]. Plastic particles are increasingly dispersed in soil due to the growing usage of plastics, which prevents their degradation and adversely affects both human health and the ecological system. The primary sources of microplastics in soil include plastic films, sludge, compost, irrigation water, and atmospheric deposition [5,6,7,8]. Polypropylene (PP), polyvinyl chloride (PVC), polyethylene (PE), and polyethylene terephthalate (PET) are common types of microplastic particles found in soil [9,10,11]. Due to their small size, microplastics are not easily visible to the naked eye and may be ingested by organisms. This allows soil microplastics to propagate through the food web and accumulate at various trophic levels, thereby posing a threat to humans and other animals [12,13,14]. In addition, microplastics in soil can alter its physicochemical properties, which may affect the entire soil ecosystem [15]. The interactions between microplastics and heavy metals or organic pollutants in soil can significantly amplify the hazards posed by microplastics to both the ecosystem and human health [16]. Therefore, it is imperative to study methods for detecting microplastics in soil. By effectively monitoring their presence and distribution, we can develop new strategies for preventing soil microplastic contamination, thereby reducing the potential threat to agriculture, ecosystems, and human health.

The traditional steps for microplastic detection primarily include sample extraction, identification, and quantification [17]. Detection methods primarily involve the extraction of microplastics from samples using physical and chemical techniques, followed by qualitative and quantitative analysis using various instruments [18]. The visual analysis approach, which uses an optical microscope to visually identify microplastics, is a widely used physical detection technique in laboratories [19]. However, identifying microplastics using this method requires operators to have extensive experience, and it is both time-consuming and inefficient [20,21]. In contrast, chemical methods primarily include Raman spectroscopy and Fourier transform infrared spectroscopy (FTIR) [22,23,24]. Asensio-Montesinos et al. identified the types of plastic materials using Raman spectroscopy [25]. Primpke et al. developed an FTIR imaging process for the automated identification and quantification of microplastic [26]. Simon et al. analyzed the material composition of plastic particles in wastewater samples using an FTIR-based imaging technique to identify microplastic particles [27]. However, these methods are destructive to the original samples and cannot capture microplastic size and shape information. Therefore, detecting soil microplastics using traditional methods is challenging, necessitating the investigation of a method for their rapid and accurate detection.

Hyperspectral imaging (HSI) is one of the most widely used techniques in various research fields [28]. Hyperspectral imaging encompasses tens or even hundreds of narrow, continuous spectral bands ranging from visible to infrared wavelengths. This technique integrates spatial and spectral information, with each pixel representing the physical properties of the material at that location. The spectral data for each pixel can be used to identify the material to which the pixel corresponds. Earlier supervised approaches like the Enhanced Multiscale Feature Fusion Network (EMFFN) [29] and hybrid attention methods such as ATN-Hybrid [30] highlight a shift toward foundation models that minimize annotation dependency and enhance generalization, paving the way for future research in cross-modal adaptability and real-time efficiency. Recent studies have developed specialized hyperspectral datasets explicitly designed for cross-scene detection tasks, as demonstrated by Liu et al. through their construction of multi-scene benchmarks featuring paired source-target domains with annotated land-cover classes to evaluate domain adaptation methods under realistic domain shifts [31].

In recent studies, Vidal et al. employed near-infrared hyperspectral imaging (HSI-NIR) to automatically and rapidly identify five common polymers and sands that constitute microplastics [32]. Moroni et al. employed hyperspectral imaging to rapidly and accurately identify two distinct microplastic polymers, confirming that this hyperspectral analysis technique is effective for detecting these polymers [33]. Xu et al. combined the HSI technique with machine learning models such as SVM, BPNN, and 1D-CNN to achieve effective classification results for detecting microplastics in contaminated agricultural soil [34]. Liu et al. utilized hyperspectral imaging techniques to detect the shape of microplastics [35]. Meanwhile, deep learning methods are widely employed in HSI classification and have recently garnered significant interest. Yurtsever et al. were the first to classify microplastics using deep learning techniques with the GoogLeNet architecture [36]. Padarian et al. utilized spectral data from unprepared soil to train a convolutional neural network (CNN) for predicting soil attributes [37]. Lorenzo Navarro et al. employed a deep learning approach to classify, identify, and count five types of plastic particles ranging from 1 to 5 mm [38]. Park et al. proposed a deep learning-based image segmentation technique to separate fluorescent microplastics from other components [39]. Wang et al. employed an enhanced Faster R-CNN model to identify microplastic particles in the marine environment [40]. Ai et al. proposed a method for recognizing soil microplastic polymers (MPPs) using convolutional neural network (CNN) and hyperspectral imaging (HSI) techniques [17]. When ample training data are available, deep learning-based techniques can yield highly accurate detection results. However, acquiring a large volume of labeled data for training models in soil microplastic detection presents a significant challenge. The field of hyperspectral image (HSI) processing has seen remarkable progress through self-supervised learning, exemplified by the HyperSIGMA foundation model, which employs masked image modeling for pre-training on large-scale data and a novel sparse sampling attention mechanism to address spectral-spatial redundancies, achieving versatility across high- and low-level tasks [41].

Although self-supervised learning has achieved great success in computer vision, applying it to the hyperspectral image classification of soil microplastics still faces several challenges. Hyperspectral images of soil microplastics are high-dimensional and have dispersed categories, making traditional image feature extraction methods difficult to directly apply. Therefore, how to effectively utilize the spatial and spectral information of hyperspectral images and design reasonable feature extraction methods to obtain more discriminative features of microplastics in the soil remains an important research direction for applying hyperspectral image classification to soil microplastic detection tasks.

This study aims to develop a Self-Supervised Hierarchical Dilated Transformer Network (SHDTNet) that integrates self-supervised contrastive learning with a hierarchical architecture to address the challenge of limited labeled samples in hyperspectral image classification. This innovation focuses on enhancing feature extraction capabilities while reducing computational complexity. This study utilizes SHDTNet to accurately identify and detect soil microplastics under low-label conditions, particularly targeting PVC, PE, PP, and PET polymers, thereby overcoming the limitations of traditional supervised methods. By precisely monitoring the spatial distribution characteristics of microplastics in the soil, this method helps to curb the threat of pollutants to agricultural production, the ecological environment, and human health, thereby providing technical support for the sustainable use of land resources and the coordinated development of regional economies.

## 2. Materials and Methods

### 2.1. Dataset and Preprocessing

**Figure 1:**
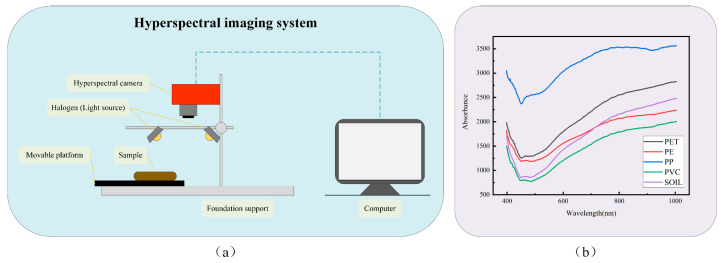
(**a**) Diagram of the hyperspectral imaging system. (**b**) Plots of average spectra of four microplastics and soil.

**Figure 2:**
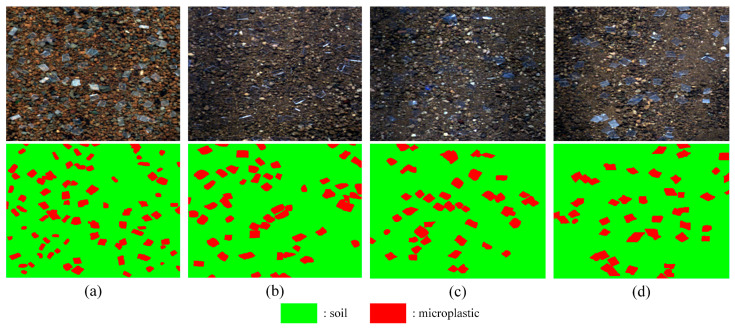
False-color composite images of four soil microplastics and visualization of reference images. (**a**) PP. (**b**) PVC. (**c**) PE. (**d**) PET.

Soil samples were first air-dried, then ground and sieved through a 2 mm metal sieve. Four types of microplastic particles (PP, PVC, PE, PET) were manually added to the soil. These plastic particles were manually cut to sizes smaller than five millimeters and added to the soil samples to simulate the distribution of microplastics in real soil. A hyperspectral camera, the SPECIM FX10e, was used to scan the soil samples. The system diagram is shown in Figure 1a and consists of a computer, a hyperspectral camera, two built-in halogen light sources, a foundation support, and a movable platform. Table 1 displays the system configuration and associated parameter settings. The system has a push-scan hyperspectral imaging function, in which soil samples are illuminated with the built-in halogen light sources and the reflected light is transmitted to the hyperspectral camera. Additionally, the SPECIM camera has a preprocessing function for black-and-white correction that uses the following equation to transform raw data to reflectance:

(1) $$Reflectance=\frac{Raw-Dark}{White-Dark}$$

where “*White*” denotes a white reference image produced by scanning a rectangular PTFE plate, “*Dark*” represents a dark reference image created by covering the lens with an opaque cover, and “*Raw*” indicates the actual data. The spectral data of the four microplastic particles and soil are presented in Figure 1b. Specific experimental procedures are detailed in the Supporting Information. Figure 2 displays the false-color composite images and reference images.

First, the original hyperspectral cube image is cropped to a spatial size of 7 × 7, which serves as the input data for the self-supervised model. The 7 × 7 patch was selected as it represents a prevalent choice in HSI classification, optimally balancing spatial context capture and computational efficiency. For soil microplastic identification, this size is particularly suitable as it aligns with the typical particle scale, providing sufficient texture information while minimizing the inclusion of irrelevant soil background noise that could hinder discrimination.

Subsequently, data augmentation techniques within the framework of self-supervised contrastive learning are employed to enhance the diversity of training samples. These techniques include spatial mirroring, random noise addition, spatial rotation, and spectral mirroring. Let I denote the input hyperspectral image data. Initially, after applying spatial mirroring, the data $\tilde{I}$ is obtained. Random Gaussian noise is then added to $\tilde{I}$ to produce the noisy data ${\tilde{I}}_{1}$. Following this, spatial rotation is applied to ${\tilde{I}}_{1}$, resulting in the rotated data {${\tilde{I}}_{1}^{\theta }$|θ∈φ}, where $\varphi =\left\{90^{\theta }\cdot t|t\in \left[0,1,2,3\right]\right\}$. Finally, in the spectral domain, a spectral mirroring operation is performed to obtain richer spectral features, resulting in the data ${\tilde{I}}_{2}^{\theta }$. Increasing the training data through spectral mirroring allows the feature extraction model to better learn the commonalities within the same class. The enhanced data ${\tilde{I}}_{2}^{\theta }$ is randomly selected as the input for the pretext task during the training phase.

### 2.2. Proposed Method

Deep learning models based on supervised learning can achieve satisfactory classification performance when sufficient labeled data is available. However, obtaining adequate labeled data in real soil microplastic application scenarios presents a significant challenge. To address this issue, a self-supervised hierarchical dilated transformer network is proposed in this paper. This model utilizes the BYOL architecture as a framework for self-supervised learning. Unlike typical self-supervised learning frameworks, BYOL does not focus on whether different samples exhibit distinct features but rather on whether the features of similar samples are also similar. There is no need to construct negative examples, and only positive examples are required to train the model, which significantly enhances the training efficiency and generalization ability of the model [42]. The BYOL model consists of an online network and a target network. The online network is composed of three stages: the encoder, the projector, and the predictor. The target network has the same architecture as the online network for the first two stages, but it lacks a predictor component and employs a different set of weights for these stages. The target network provides regression targets to train the online network, which is parameterized by an exponential moving average of the online parameters. Given a target decay rate τ ∈ [0,1], the following updates are performed after each training step:ξ←τξ+(1−τ)θ.

#### 2.2.1. Overview of Self-Supervised Hierarchical Dilated Transformer Networks

**Figure 3:**
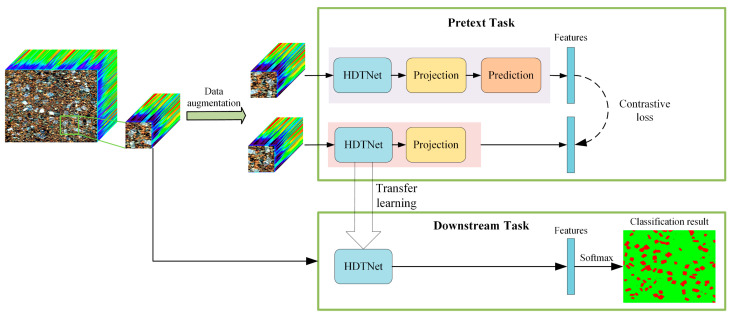
Flowchart of the proposed method for detecting soil microplastics.

The flow of the proposed method for detecting soil microplastics is illustrated in Figure 3. First, in the pretext task, a self-supervised hierarchical dilated transformer network is employed to extract deep features from hyperspectral images of soil microplastics. Subsequently, the parameters of the pre-trained hierarchical dilated transformer model are transferred to the downstream classification task, where the model parameters are fine-tuned using a small amount of labeled data. Finally, soil microplastics are identified and detected.

The pretext task uses unlabeled augmented images to learn features. By employing a hierarchical dilated transformer network, the deep features *f*_1_ and *f*_2_ of the unlabeled samples *x_1_* and *x_2_* can be obtained. The training objective is to maximize the distance between different features while minimizing the distance between similar features, thereby learning meaningful deep features from unlabeled examples. The loss function aims to minimize the discrepancy between the output of the prediction in the online network and the output of the projection in the target network. The contrastive loss is calculated as follows:

(2) $$L≜{\left\|\bar{Y}-\bar{Y^{\prime }}\right\|}_{2}^{2}=2-2\cdot \frac{\langle Y,Y^{\prime }\rangle }{{\left\|Y\right\|}_{2}\cdot {\left\|Y^{\prime }\right\|}_{2}}$$

where Y is the output of the online network prediction and $Y^{\prime }$ is the output of the target network projection. At the end of the training process, only the encoder component is retained, while all other components are discarded.

Lastly, the downstream classification network receives the encoder parameters that were learned during the pretext task, and a small amount of labeled data is used to train the hierarchical dilated transformer model. The model parameters are fine-tuned to address the classification problem by minimizing the classification objective function:

(3) $$Loss=-\frac{1}{C}\sum_{i=0}^{C}\left(y_{i}\log\left({\hat{y}}_{i}\right)+\left(1-y_{i}\right)\log\left(1-{\hat{y}}_{i}\right)\right)$$

where *C* is the total number of classes, $y_{i}$ is the true value, and ${\hat{y}}_{i}$ is the predicted label.

#### 2.2.2. Hierarchical Dilated Transformer Network (HDTNet)

**Figure 4:**
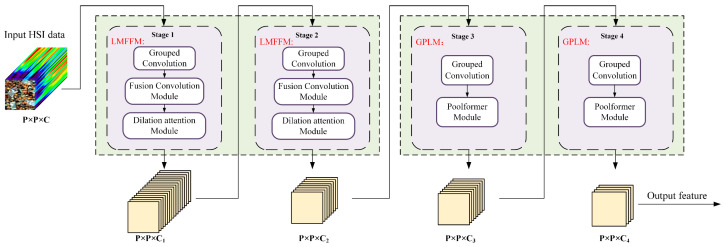
Overall structure of the HDTNet model.

The DilateFormer model was introduced by Jiao et al. in 2023 [43], The proposed method utilizes multiscale dilated attention to model the interactions of localized and sparse patches within a sliding window. It incorporates a pyramid structure with global multi-headed self-attention blocks stacked at the low-level stages to address the redundancy in modeling shallow global dependencies inherent in Vision Transformers (ViTs), while reducing FLOPs by 70% compared to other state-of-the-art models from the same period, thus achieving comparable performance. Building on this model, an enhanced HDTNet model was developed for the detection and identification of soil microplastics. The overall structure of the model is illustrated in Figure 4. This model generates feature maps at various scales using a four-stage framework, with the Local Multi-Scale Feature Fusion Module (LMFFM) applied in the first two stages and the Global Pooling Lightweight Module (GPLM) used in the last two stages. Given an input HSI patch cube $X\in R^{P\times P\times C}$*,* where P is the spatial size of the input HSI patch and C is the number of bands in the entire HSI. The entire patch is input into a four-stage feature extraction framework, where the four stages produce corresponding feature maps with dimensions *P × P × C*_1_, *P × P × C*_2_, *P × P × C*_3_, and *P × P × C*_4_, respectively. In our model, the parameters for these four stages are set to {256, 128, 128, 64}, respectively. This hierarchical model structure, which employs dimensionality reduction, helps to decrease resource consumption while effectively capturing useful features [44].

(1) LMFFM: The Local Multi-Scale Feature Fusion Module (LMFFM) includes grouped convolution, a fusion convolution module, and a dilation attention module. The first two layers utilize the LMFFM primarily to extract local feature information from soil microplastic hyperspectral images.

Recent research has incorporated grouped convolution into the field of hyperspectral image (HSI) classification. Many state-of-the-art models employ convolutional neural networks (CNNs) in transformers to extract local features from images. In this paper, ordinary CNNs are replaced with grouped convolution to enhance the extraction of feature representations for soil microplastics. Grouped convolution has fewer parameters and is less prone to overfitting compared to standard CNNs. Additionally, it enables the extraction of discriminative information from the subsequent subchannels of feature mappings. Consequently, grouped convolution is more effective at capturing local feature information. The proposed model divides the number of input channels into 16 groups.

The detailed structure of the fusion convolution module and the dilation attention module is as follows:

(a) Fusion Convolution Module: The fusion convolution module was introduced by Zhang et al. in 2023 [45]. The fusion convolution module was introduced to address the issue of slow processing speeds in the early stages of feature extraction. By employing fused convolutional modules in these initial stages of Vision Transformers (ViTs), the efficiency of the ViT model is enhanced. Applying the fusion convolution module to hyperspectral soil microplastic detection improves the extraction of spatial information from hyperspectral images, particularly when dealing with the irregular distribution of soil microplastics, thereby increasing the accuracy of detection. The details of the fusion convolution module are described in the Supporting Information.

(b) Dilation Attention Module: The dilation attention module consists of alternating layers of Multi-Headed Dilated Attention (MHDA) and Multi-Layer Perceptron (MLP). The structure of the dilation attention module is illustrated in Supporting Information Figure S2a. MHDA is an enhancement of Multi-Headed Self-Attention (MHSA), a mechanism designed to establish global remote dependencies and extract important features. MHSA increases the diversity of the feature subspace by parallel processing inputs and projecting them into multiple feature subspaces without additional computation time, thereby improving computational efficiency [46]. For details on the specific process, refer to the Supporting Information.

**Figure 5:**
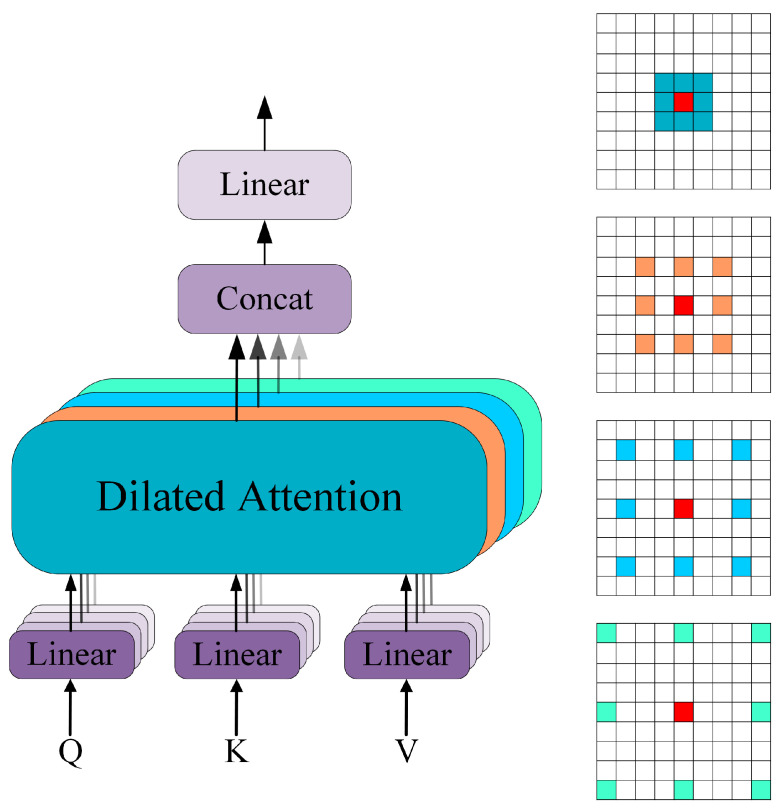
Schematic diagram of Multi-Headed Dilated Attention (MHDA).

To integrate features of different scales, dilation convolution is introduced in the Multi-Headed Dilated Attention (MHDA) module to extract multi-scale semantic information. The specific structure of the MHDA module is illustrated in Figure 5. Features are distributed across four different heads, with dilation convolution applied to each head using varying dilated rates. The dilated rates are set to $\left\{r|r\in \left[1,2,3,4\right]\right\}$, enabling the extraction of features at different scales across the heads.

(2) GPLM: The GPLM consists of grouped convolution and a Poolformer module. The latter two layers of the model use the GPLM primarily for extracting global feature information from hyperspectral images of soil microplastics. The Poolformer module is described as follows.

(a) Poolformer Module: The Poolformer module was introduced by Yu et al. in 2021 [47]. The structure of the Poolformer module is illustrated in Supporting Information Figure S2b. The Poolformer module employs a simple pooling operation as a token mixer. This approach facilitates the extraction of global features while simultaneously reducing computational parameters and enhancing computational efficiency compared to the self-attention mechanism of the original Transformer model. Hyperspectral images, which contain rich spectral features, lead to a large number of computational parameters and less efficient model training compared to ordinary RGB images. Integrating the Poolformer module into the latter two stages of this model creates a more lightweight architecture, thereby enabling faster soil microplastic detection.

## 3. Results and Discussion

### 3.1. Evaluation Indicators

To quantitatively assess the classification performance of the improved model for soil microplastics, we used overall accuracy (OA), average accuracy (AA), and the Kappa coefficient as evaluation metrics. These metrics were computed based on the Confusion Matrix.

OA represents the proportion of correctly predicted samples out of the total number of samples. The calculation formula is as follows:

(4) $$OA=\frac{TP+TN}{TP+FN+FP+TN}$$

where *TP* represents a positive sample correctly classified by the model, *FN* denotes a positive sample incorrectly classified by the model, *TN* signifies a negative sample correctly classified by the model, and *FP* refers to a negative sample incorrectly classified by the model.

AA represents the average classification accuracy across all categories and is calculated as follows (for binary classification):

(5) $$AA=(\frac{TP}{TP+FN}+\frac{TN}{FP+TN})/2$$

Kappa is a statistical measure used to evaluate the agreement between ground truth maps and classification maps, and is calculated using the following formula:

(6) $$Kappa=\frac{p_{0}-p_{e}}{1-p_{e}}$$

where $p_{0}$ represents the value of OA, and $p_{e}$ is calculated as

(7) $$p_{e}=\frac{\left(TP+FP\right)\left(TP+FN\right)+(TN+FN)(FP+TN)}{{\left(TP+FN+FP+TN\right)}^{2}}$$

### 3.2. Model Training

All experiments were conducted using the PyTorch 1.12 deep learning framework. The specific computer configurations are detailed in Table 2. For updating the training parameters, a stochastic gradient descent (SGD) optimizer was employed with a minimum batch size of 128, and momentum and weight decay values of 0.9 and 0.0001, respectively. τ is set to 0.996. The total number of iterations was set to 300, with 100 iterations dedicated to the pretext task and 200 iterations to the downstream classification task. In the downstream classification task, the learning rate was maintained at a constant value of 0.001. For training, 10% of the labeled samples were randomly selected for validation, and the remaining 80% were used for testing.

### 3.3. Comparison of Detection Effects

To validate the effectiveness of the added modules, experiments were conducted on the PP dataset. Table 3 presents the classification accuracy of the proposed method with and without these modules. It is evident that the proposed approach achieves higher accuracy when incorporating the fusion convolution module. The absence of this module impairs the method’s ability to effectively extract spatial information features from hyperspectral images. Given that microplastics in the soil environment are characterized by irregular spatial distribution, the fusion convolution module proves essential. The dilation attention module primarily extracts multi-scale features from hyperspectral images, a critical aspect of the feature extraction process. As shown in Table 3, detection accuracy is lowest when the dilation attention module is not utilized. Furthermore, the removal of the self-supervised BYOL model results in a significant decline in performance when training samples are limited. This decline occurs because self-supervised contrastive learning improves generalization. Additionally, replacing the conventional Multi-Headed Self-Attention (MHSA) with the Poolformer module considerably reduces training time and improves detection rate, while still maintaining high accuracy.

**Figure 6:**
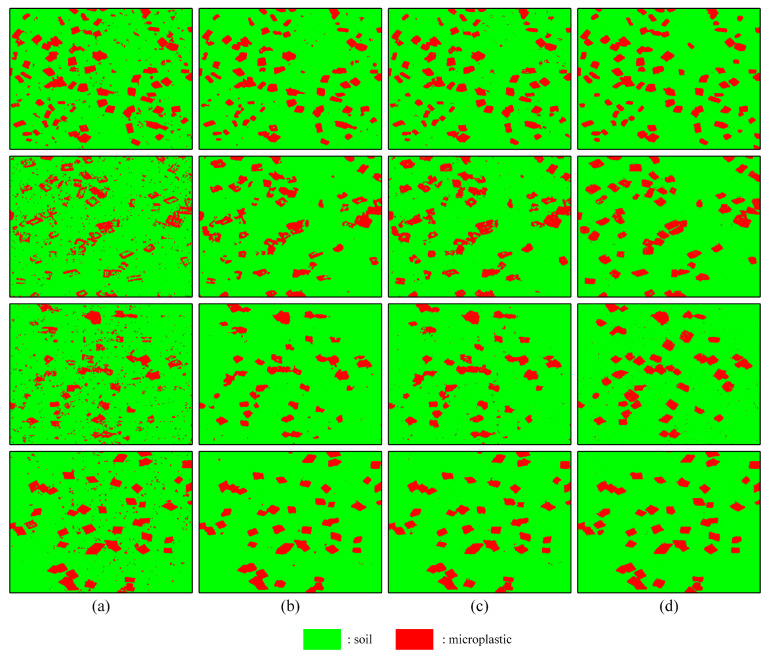
Classification maps obtained with different methods on the four datasets. (**a**) SVM. (**b**) 3DCNN. (**c**) SpectralFormer. (**d**) Proposed.

To illustrate the sensitivity of the proposed model in identifying different types of microplastics, Figure 6 and Table 4 provide a comparative analysis between models. The support vector machine (SVM) method proves ineffective in detecting soil microplastics, resulting in “pretzel” noise within the classification diagram. Additionally, due to the spectral similarities between PVC and PE microplastics and the soil, the previous three models fail to adequately recognize these two types of microplastics, leading to significant missed detections. This shortcoming is detrimental to accurately assessing the distribution and concentration of microplastics in the soil. On the self-constructed dataset of four soil microplastic types, the proposed SHDTNet achieved an overall accuracy (OA) exceeding 98%, outperforming all three baseline models. This demonstrates that the SHDTNet model is superior at detecting soil microplastics. In contrast, the other three network models suffer from misdetection issues. The enhanced SHDTNet model provides a robust solution for the efficient and precise detection of soil microplastics.

At the same time, the proposed HDTNet is a hierarchical deep network, with each layer containing computationally intensive components. In particular, the self-attention mechanism in the dilated Transformer module has a computational complexity proportional to the square of the input sequence length. For hyperspectral images, which involve the fusion of spatial and spectral information, the sequence length (or the number of pixels) is typically very large, resulting in significant computational overhead. Moreover, although the hierarchical structure effectively reduces dimensionality and decreases the total number of parameters, its “staged processing” approach itself introduces additional data transfer and coordination overhead, making the training process indeed longer compared to a single-stage flat network.

Despite the promising results, it is crucial to acknowledge the limitations of this study to provide a balanced perspective and guide future research. First, the training and validation of our model relied on an artificial dataset comprising soil samples spiked with known microplastics. While this approach ensures controlled concentrations for method development, it may not fully capture the complex aging, weathering, and biofouling characteristics of microplastics in naturally contaminated environmental samples, potentially affecting the model’s real-world detection accuracy. Second, our experiments were conducted under specific and relatively homogeneous laboratory conditions. The model’s performance remains untested across the vast spectrum of natural variability, such as different soil types, varying moisture content, and inherent environmental heterogeneity, which could significantly influence spectral responses and detection efficacy. Third, the current validation was performed on an internal dataset. The absence of external validation using independent, publicly available datasets limits our ability to conclusively demonstrate the model’s generalizability to other scenarios and sampling protocols. Finally, the performance comparisons with existing methods, while indicative of superior accuracy, were not supplemented with statistical significance tests. Future work must incorporate rigorous statistical analyses to fortify the robustness of these comparative claims. Addressing these limitations will be the primary focus of our subsequent research efforts.

## 4. Conclusions

In this paper, we propose a soil microplastic identification technique based on a self-supervised contrastive learning classification method. An enhanced deep learning model is developed by integrating self-supervised contrastive learning with hyperspectral imaging to address the challenge of insufficient labeled samples in soil microplastic scenarios. On a controlled, artificially prepared dataset, this approach achieves accurate detection of microplastic particles made from four common polymers (PP, PVC, PE, PET). Experiments demonstrate that the accuracy remains as high as 98% with limited training samples, suggesting its potential to reduce issues like missed detections and errors in similar controlled settings. However, these findings are derived from an artificial dataset, and further validation in real-world soil conditions is necessary to assess broader applicability. The study provides a methodological foundation for future large-scale assessments of soil microplastic concentrations, though generalization to natural environments requires additional research.

Although the self-supervised contrastive learning method used in this study can to some extent address the difficulty of labeling hyperspectral images of soil microplastics, it requires a large amount of training data and high computational costs during the contrastive learning pretraining phase. Therefore, the next step is to adopt other more advanced self-supervised learning methods for the task of hyperspectral soil microplastic identification, such as masked autoencoders, to achieve more efficient self-supervised hyperspectral soil microplastic recognition.

## Figures and Tables

**Table 1:** System configuration of the hyperspectral system.

Configuration	Parameters
The operating system of the computer	Windows 11 Education, 64-bit
The processor of the computer	Intel(R) Core(TM) i7-8750H
Spectral coverage	400~1000 nm
Band number	224
Rated power of the halogen lamp	Single not less than 35 W

**Table 2:** Computer configuration for the experiment.

Configuration	Version
System	Ubuntu 20.04.1, 64-bit
Processor	Intel(R) Xeon(R) Silver 4210R CPU @ 2.40GHz
GPU	NVIDIA GeForce RTX3090
Language	Python 3.7.10
CUDA	11.7

**Table 3:** Comparative results of ablation experiments on PP dataset (%).

BYOL	Fusion Convolution Module	Dilation Attention Module	Poolformer Module	OA	AA	Kappa	Train Time (min)
-	√	√	√	98.65	96.74	94.39	-
√	-	√	√	98.31	95.90	92.95	-
√	√	-	√	97.78	95.36	90.86	-
√	√	√	-	99.01	98.19	95.95	254
√	√	√	√	99.35	98.54	97.33	239

**Table 4:** Classification results of different methods on the four datasets (%).

Database	Indexes	SVM [48]	3DCNN [49]	Spectral Former [50]	Proposed
PP	OA	94.03	97.1	97.46	99.35
	AA	85.48	93.36	94.32	98.54
	Kappa	78.5	87.90	89.45	97.33
PVC	OA	89.37	93.69	93.68	98.33
	AA	68.58	85.47	86.77	95.57
	Kappa	46.34	73.43	74.11	91.76
PE	OA	91.89	94.86	95.02	98.05
	AA	71.27	82.40	84.84	93.82
	Kappa	51.23	71.94	74.00	90.12
PET	OA	96.8	97.94	98.12	98.82
	AA	90.3	93.67	94.61	97.28
	Kappa	84.10	89.90	90.88	94.37

## Data Availability

Data will be made available on request.

## Supplementary Materials

The following supporting information can be downloaded at: https://www.mdpi.com/article/10.3390/s25216517/s1. Figure S1. (a) Fused convolution module. (b) SE module. Figure S2. (a) Dilation attention module. (b) Poolformer module. (c) MLP structure diagram.
