# Supplementary material for: Self-Supervised Hierarchical Dilated Transformer Network for Hyperspectral Soil Microplastic Identification and Detection
Source: Sensors (Basel). 2025 Oct 22;25(21):6517. doi: 10.3390/s25216517 (PMC12610989; doi:10.3390/s25216517)
Supplement: Supplementary file 1 [file sensors-25-06517-s001.zip › sensors-3905612-supplementary.pdf]

Self-supervised hierarchical dilated transformer network for hyperspectral soil microplastic identification and detection

Peiran Wang [1,2], Xiaobin Li [3], Ruizhe Zhang [4], Qiongchan Gu [2,4], Lianchi Zhang [5,*], Jiangtao Lv [2,4,*]

[1] School of Intelligent Sensing and Optoelectronic Engineering, Northeastern University at Qinhuangdao, Qinhuangdao 066004, China; wangpeiran2004@126.com
[2] School of Control Engineering, Hebei Key Laboratory of Micro-Nano Precision Optical Sensing and Measurement Technology, Northeastern University at Qinhuangdao, Qinhuangdao 066004, China; guqiongchan@neuq.edu.cn; lvjiangtao@neuq.edu.cn
[3] Water Resources and Water Conservation Development Center of Chengde, Chengde 067000, China; lxb13703140653@163.com
[4] College of Information Science and Engineering, Northeastern University, Shenyang 110004, China; zhangrz5@mails.neu.edu.cn
[5] FAW Car Co., Ltd, Changchun 130012, China; zhanglianchi@faw.com.cn
* Correspondence: zhang_lianchi@163.com; lvjiangtao@neuq.edu.cn

1. Experimental procedures

The experimental procedure was as follows: (1) Microplastic samples were prepared from four polymers. The computer was connected to the remainder of the hyperspectral imaging system via the LUMOS canner spectrometer software, which also managed the motion of the picture acquisition stage. The system's initial settings included a 10 ms camera exposure time, a 10 ms/s moving platform speed, a 15 cm distance between the lens and the sample, and spectral band selection of 224. (2) Due to environmental disturbances and instrumental vibrations during the acquisition process, black and white correction eliminated the disturbances and reduced the noise signal. Thus, prior to acquiring samples, the original picture must be rectified using a standard all-white reference image and an all-black reference image; the corrected image can be used with the following equation：

$$\text{Reflectance} = \frac{\text{Raw}-\text{Dark}}{\text{White}-\text{Dark}} \tag{1}$$

(3) Distribute the samples evenly and position them at the center of the platform. Subsequently, the hyperspectral images of the samples were sequentially acquired while the electronically controlled platform was moved. By repeating step (3), hyperspectral images of four types of soil microplastics were collected. Equation (1) was then used to adjust the collected data. Following image collection, the discarded experimental materials were recycled. The spectral data for the four microplastic types and soil are

presented in Figure 1(b). Figure 2 presents the false-color composite images and reference images.

2. Introduction to the DilateFormer model

The first two stages of the model utilize the proposed Multiscale Dilation Attention (MSDA), while the final two stages employ the conventional Multi-headed Self-Attention (MHSA). To effectively reduce redundancy in the self-attention mechanism and efficiently aggregate semantic multiscale information without necessitating complex operations or incurring additional computational costs, the DilateFormer model incorporates localization and sparsity considerations at the shallow level of the self-attention mechanism. It provides a robust and versatile representation suitable for various visual tasks, thereby enhancing detection accuracy. In this study, the network serves as the base model.

3. The fusion convolution module

The structure of the fusion convolution module is illustrated in Figure 1(a). The number of channels is doubled using a 3×3 convolution operation. The SE module is illustrated in Figure S-1(b). The SE module comprises a global pooling operation, a fully connected layer, a ReLU activation function, another fully connected layer, and a sigmoid activation function. Finally, the number of channels is reverted to its original count using a 1×1 projection layer, which facilitates the subsequent addition of inputs and outputs.

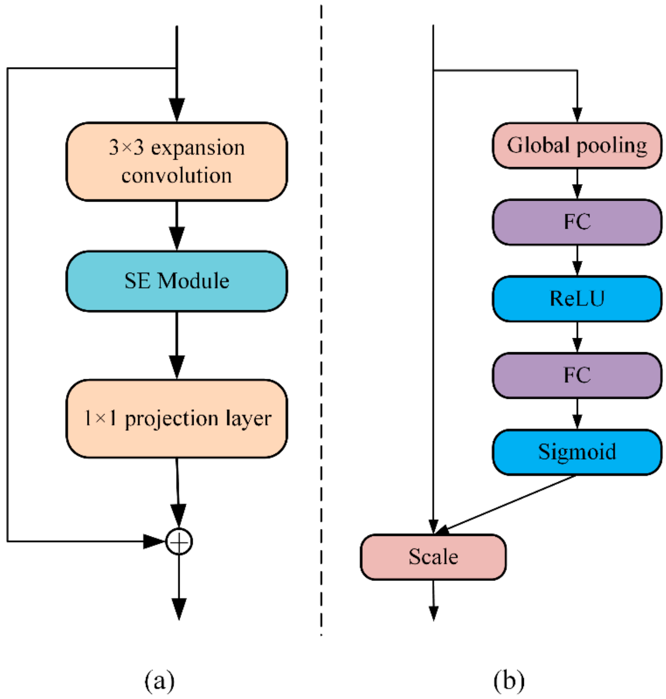

Figure S-1. (a) Fused convolution module. (b) SE module.

4. Dilation Attention Module Details

Specifically, the input $X \in R^{C\times H\times W}$ is first mapped to three different matrices by linear transformations as follows: query ($Q \in R^{C\times H\times W}$), key ($K \in R^{C\times H\times W}$) and value($V \in R^{C\times H\times W}$):

$$\mathrm{Q} = \mathrm{XW^Q}, \mathrm{K} = \mathrm{XW^K}, \mathrm{V} = \mathrm{XW^V} \quad (2)$$

where W^Q, W^K and W^V are trainable parameter matrices. Q, K and V are then divided by 4 along the dimension axis as follows:

$$\mathrm{Q} = \{\mathrm{Q_1}, \mathrm{Q_2}, \mathrm{Q_3}, \mathrm{Q_4}\} \quad (3)$$

$$\mathrm{K} = \{\mathrm{K_1}, \mathrm{K_2}, \mathrm{K_3}, \mathrm{K_4}\} \quad (4)$$

$$\mathrm{V} = \{\mathrm{V_1}, \mathrm{V_2}, \mathrm{V_3}, \mathrm{V_4}\} \quad (5)$$

where $Q_i, K_i, V_i \in R^{s\times(d/4)}$. The dilation convolution with different dilated rates is then used for each Q_i to obtain Q_{ci}, as follows:

$$\mathrm{Q_{ci}} = \mathrm{DilatedConv}(\mathrm{Q_i}) \quad (6)$$

where $DilatedConv(\cdot)$ denotes dilation convolution. Next, a scaled softmax function calculates the attention score for each i:

$$\mathrm{Z_i} = \mathrm{Attention}(\mathrm{Q_{ci}}, \mathrm{K_i}, \mathrm{V_i}) = \mathrm{Softmax}(\frac{\mathrm{Q_{ci}K_i^T}}{\sqrt{\mathrm{d}}})\mathrm{V_i} \quad (7)$$

The final output is the projection after splicing all $Z_i \in R^{s\times(d/n)}$, which can be expressed as

$$\text{Output} = \text{Concat}(Z_1, Z_2, Z_3, Z_4)W \tag{8}$$

where W denotes the output projection matrix.

The MLP is a two-layer linear projection that extracts the feature representation using a Gaussian Error Linear Unit (GELU) nonlinear in the center. The structure of MLP is shown in Figure S-2 (c). The MLP module can be summarized as follows

$$\text{MLP}(X) = \text{FC}_2(\text{GELU}(\text{FC}_1(X))) \tag{9}$$

The linear projection part is realized by the Fully-connected Layer. GELU is a linear projection function often applied to ViT models and defined as follows:

$$\text{GELU}(X) = X\emptyset(X) = \frac{X}{2}[1 + \text{erf}(\frac{X}{\sqrt{2}})] \tag{10}$$

where $\emptyset(X)$ is the standard Gaussian cumulative distribution function, $erf(X) = \int_0^X e^{-t^2}\, dt$. In the transformer block, the MLP layer and MHSA module are positioned after the LayerNorm layer. Additionally, residual connections are utilized to mitigate the overfitting issues commonly associated with deep learning techniques [1].

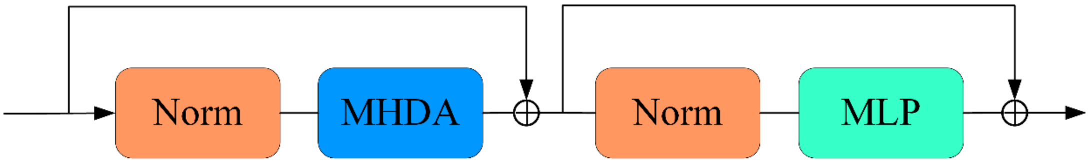

(a)

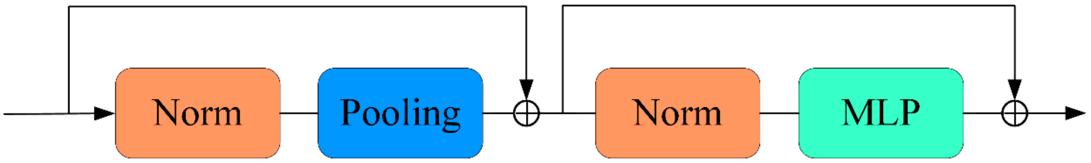

(b)

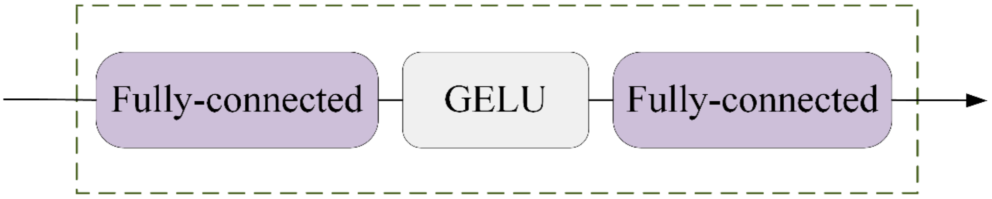

(c)

Figure S-2. (a) Dilation attention module. (b) Poolformer module. (c) MLP structure diagram.

Reference

[1] He, K., Zhang, X., Ren, S., Sun, J., 2016. Deep Residual Learning for Image Recognition, in: 2016 IEEE Conference on Computer Vision and Pattern Recognition (CVPR). Presented at the 2016 IEEE Conference on Computer Vision and Pattern Recognition (CVPR), pp. 770–778. https://doi.org/10.1109/CVPR.2016.90
